# Psychological interventions for maternal depression among women of African and Caribbean origin: a systematic review

**DOI:** 10.1186/s12905-021-01202-x

**Published:** 2021-02-26

**Authors:** Dung Ezekiel Jidong, Nusrat Husain, Ayesha Roche, Grace Lourie, Tarela J. Ike, Maisha Murshed, Miriam S. Park, Haruna Karick, Zubairu K. Dagona, Juliet Y. Pwajok, Anil Gumber, Christopher Francis, Pam P. Nyam, Shadrack B. Mwankon

**Affiliations:** 1 Department of Psychology, Nottingham Trent University, 50, Shakespeare Street, Nottingham, NG1 4FQ UK; 2 University of Manchester, Manchester, UK; 3 Teesside University, Middlesbrough, UK; 4 University of Jos, Jos, Nigeria; 5 Sheffield Hallam University, Sheffield, UK; 6 Federal University Oye-Ekiti, Oye-Ekiti, Nigeria

**Keywords:** African, Culture, Caribbean, Depression, Psychological intervention, Maternal, Mothers, Women

## Abstract

**Background:**

Maternal depression is a leading cause of disease burden for women worldwide; however, there are ethnic inequalities in access to psychological interventions in high-income countries (HICs). Culturally appropriate interventions might prove beneficial for African and Caribbean women living in HICs as ethnic minorities.

**Methods:**

The review strategy was formulated using the PICo (Population, phenomenon of Interest, and Context) framework with Boolean operators (AND/OR/NOT) to ensure rigour in the use of search terms (“postpartum depression”, “maternal depression”, “postnatal depression”, “perinatal depression” “mental health”, “psychotherapy” “intervention”, “treatment”, “black Caribbean”, “black African”, “mothers” and “women”). Five databases, including Scopus, PsycINFO, Applied Social Science Index and Abstracts (ASSIA), ProQuest Central and Web of Science, were searched for published articles between 2000 and July 2020. 13 studies met the inclusion criteria, and the relevant data extracted were synthesised and thematically analysed.

**Results:**

Data syntheses and analyses of included studies produced four themes, including (1) enhance parenting confidence and self-care; (2) effective mother–child interpersonal relationship; (3) culturally appropriate maternal care; and (4) internet-mediated care for maternal depression.

**Conclusion:**

In the quest to address maternal mental health disparities among mothers of African and Caribbean origin in HICs, the authors recommend culturally adapted psychological interventions to be tested in randomised control trials.

**Supplementary Information:**

The online version contains supplementary material available at 10.1186/s12905-021-01202-x.

## Background

Women of African and Caribbean origins in High-Income Countries (HICs) are particularly vulnerable to the high risk of maternal mortality than women of other ethnic backgrounds. In the United Kingdom (UK), maternal deaths due to mental and physical health complications among African and Caribbean women are 41.1 and 62.2 per 100,000 pregnancies compared with 11.1 per 100,000 pregnancies of their white women counterparts [1, 2]. Similarly, the prevalence of maternal mental health varies significantly by ethnic groups. For example, 29.3% of African and Caribbean women are more likely to suffer from mental health problems compared to 20.9% of White British women [3]. Evidence suggests that women from White family background have better access to culturally appropriate mental healthcare for maternal depression compared to the women of African and Caribbean origins [4, 5], who are most likely to be ignored, not followed up about their current or past maternal health distress, and not offered any form of treatment for postnatal depression compared with their white counterparts [6].

According to the World Health Organisation in 2019, depression during or after childbirth affects approximately 13% of women during the postnatal period and 10% of women during pregnancy [7]. Mothers with maternal depression feel multiple symptoms such as the sense of hopelessness, withdrawn behaviours, severe mood swings, lack of concentration, and unexplainable pains and fatigue [8, 9]. Maternal depression has potential risks and fatal implications for the mother, child and the entire family [5, 8, 9]. The attachment between a mother and child is critical for the child's physical and cognitive development at both present and later life [10, 11]. This suggests that maternal depression can have multigenerational adverse effects.

However, little is known about the maternal mental health of African and Caribbean women. A systematic review has shown that women from ethnic minority groups are at a higher risk of developing maternal depression [12]. This may be due to racial discrimination, social deprivation and lack of culturally sensitive interventions [13, 14]. Risk factors for maternal depression in ethnic minority women could be explained by the critical race theory which explores the unequal distribution of resources, diagnosis and treatment of mental health conditions, and societal and political manifestations as the result of racial stratification [15]. The current mental healthcare systems do not serve the needs of depressed African and Caribbean women effectively [12]. In the UK, mental health professionals predominantly from the white British background have reported a lack of confidence and inadequate cultural competence training and their inability to detect perinatal depression in women of African origin [16]. Thus, there are so few practitioners of African origin, and therefore, lack ‘insider knowledge’ relating to Black, African and Caribbean cultures [16].

The National Institute for Health and Care Excellence (NICE) guideline for postnatal depression recommends cognitive behaviour therapy and interpersonal therapy as first-line treatment [2]. Despite the evidential benefits of interventions in preventing or managing maternal mental health, culturally sensitive interventions are sparse in targeting women of African and Caribbean origin. A recent systematic review has suggested that culturally adapted interventions have better outcomes compared to routine treatment [17]. One study delivered a culturally adapted group psychological intervention for postnatal depression for the British South Asian mothers [18]. The intervention participants reported an overall positive change in their attitudes, behaviour and self-confidence [18]. The study also suggests the acceptability and feasibility of interventions of this kind.

Africans and Caribbean people’s experiences are unique or different from mainstream culture and other minority ethnic people in HICs. For example, the adversities and legacies of slavery, colonialism, and racism potentially impact not only mental health issues but also social realities and general worldviews [19].

Therefore, there is a clear need to identify interventions that specifically address African and Caribbean mothers’ psychological needs. The purpose of this study is to review the existing interventions for maternal depression aimed at women of African and Caribbean ethnicity living in HICs.

## Methods

### Protocol/search strategy

The review protocol was developed and registered with the International Prospective Register of Systematic Reviews (PROSPERO; CRD42019149392). The review used the Preferred Reporting Items for Systematic Reviews and Meta-Analyses (PRISMA) to ensure methodological rigour and global best practice for systematic review [20]. Utilising Boland, Cherry and Dickson’s [21] model, a protocol outlining the inclusion criteria and specific methods of analyses postulated in advance of undertaking this review [21]. All records were collected on RefWorks, a bibliographic tool used in research to develop personalised databases.

### Inclusion and exclusion criteria

The review inclusion criteria were: (1) study population of women and mothers, self-identified as Black or having African or Caribbean origin; (2) providing intervention in the form of psychological therapy, educational programs or health visitor interventions; (3) studies which demonstrate maternal depression and well-being outcomes and/or child development/ behaviour/well-being outcomes; and (4) studies using either qualitative or quantitative methods with pre- and post-intervention measures or randomised controlled trials.

Papers were excluded if: (1) the study population had a majority of Caucasian participants or contained ethnic minority groups that were not inclusive of participants with Black, African or Caribbean origin; (2) studies where interventions did not focus on maternal well-being and depression; (3) studies that only measured the outcomes of child development/behaviour/well-being; or (4) studies with correlational design, with an emphasis on the prevalence of depression only without any intervention tested or provided.

### Information sourcing

The strategy adopted to formulate the review was the PICo model (Population [Black, African and Caribbean mother/child], the phenomenon of Interest [depression], Context [maternal mental health and child well-being]) [22]. The Boolean operators (AND/OR/NOT) were also used for strategic search [23]. The search terms include “postpartum depression”, “maternal depression”, “postnatal depression”, “intervention”, “treatment”, “program*”, “black Caribbean”, “black African”, “black”, “Afro-Caribbean”, “African” and “Caribbean” “mothers” and “women” to search five databases (Scopus, PsycINFO, ProQuest Central, Applied Social Science Index and Abstracts (ASSIA) and Web of Science) for published studies between 2000 and July 2020.

### Screening and selection

The search yielded a total of 553 records, of which duplicates removed were n = 259. Two reviewers screened the available titles and abstracts by applying the inclusion–exclusion criteria. After full texts were screened, 13 studies were selected for the systematic literature review. Any discrepancies between the two reviewers were resolved through discussion with a third reviewer.

### Risk of bias assessment

The present review used the Standard Quality Assessment Criteria (QualSyst Tool) for the risk of bias evaluation [24] (see the Additional file 1 table showing the risk assessment of each study). All studies included in the review showed a low risk of bias. Majority of the studies used control groups (collaborative care or treatment as usual). Due to mothers’ vulnerable nature with maternal depression, none of the studies withheld treatment as usual from participants. Most studies included in the review used randomisation to allocate participants to the different arms of the trial. The majority of the studies provided limited information on the process of blinding or whether this occurred.

However, one study reported proximal outcomes and related this to the small sample size [25], and another study only supported the conclusions made due to lack of significance in the results [26]. The majority of research were of small sample size pilot studies testing the feasibility of maternal interventions. Attrition is a challenge in these types of studies where the sample populations are under-treated. For example, one study had only 3% attendance in the control group compared to 83% attendance in the intervention group [27]. The majority of studies recruited non-treatment seeking participants which may reflect the challenges with engagement and one internet-based intervention study examined mothers’ intention to seek treatment for maternal depression.

### Strategy for data synthesis

The findings from the various studies were thematically synthesised into a qualitative narrative addressing the research question. Therefore, the conceptual framework of synthesis was adopted [28]. Each source was critically analysed and evaluated by major themes in terms of strengths, weaknesses and critical gaps using the synthesis matrix. The synthesis was conducted in four stages: (1) implementation of a relevant literature search; (2) identified key ideas and elements; (3) organised the key ideas and elements; (4) synthesised data and built a case for new research intervention. An ‘index card’ like method was employed to identify and organise the key ideas and elements from stages 2 and 3. Each pile or category of organised ideas were re-arranged into (a) a logical flow of information; (b) compare and contrast; (c) critique; and the (d) postulation of an alternative model for future interventions research aimed at African and Caribbean mothers/women.

## Results

The extracted data were synthesised into the following four themes: (1) enhance parenting confidence and self-care; (2) effective mother–child interpersonal relationship; (3) culturally appropriate maternal care; and (4) internet-mediated care for maternal depression.

**Fig. 1:**
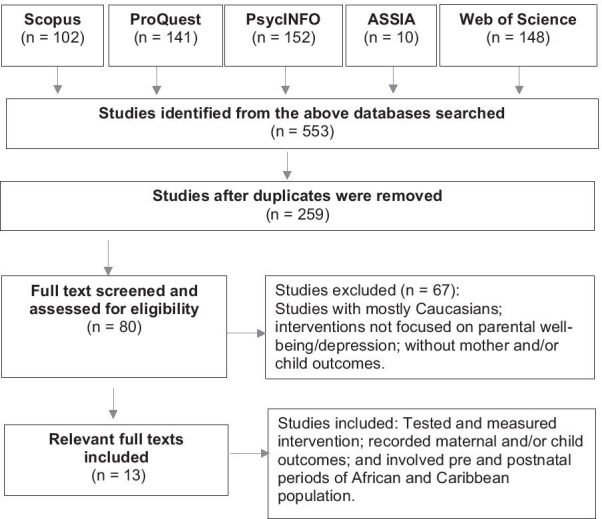
Showing trajectory of the literature review process

The flow diagram in Fig. 1 showed a trajectory of the literature that was synthesised. Subsequently, the characteristics of studies that met the inclusion criteria (see Table 1) illustrate data extraction from selected studies.

**Table 1:** Characteristics of selected studies

Author(s)	Aims	Sample population	Main findings	Conclusion	Limitations
Boyd et al. [27]	Randomised controlled trial to describe the adaptations of a parenting group intervention for social media, examine the feasibility, accessibility and initial outcomes of the adapted interventions for mothers with postpartum depressive symptoms	24 mothers with a child between 1 and 3 months old recruited from three urban paediatric primary care clinics, USA	Mothers in the social media intervention group showed a greater reduction in depression than the mothers in the in-person group (F(3,15) = 8.27, 95% CI [− 18.0 to − 2.2], *p* < 0.01) The experimental group also showed a greater increase in parenting competence than the control group Average attendance was 83%, and average participant commenting on the group page was 73% for the social media intervention group, compared to in-person group average attendance of 3%	The study findings suggest the feasibility and benefit of delivering a parenting intervention via social medical for mothers with postpartum depression	The in-person group has less contact shown by low attendance rate, which could account for the lack of improvement. The study did not gather information regarding whether the participants were in behavioural treatment at the time of the study
Crockett et al. [26]	Randomised controlled trial to examine the initial acceptability, feasibility and effectiveness of the ROSE program a brief interpersonally based intervention compared to treatment as usual (TAU)	36 low-income women who were between 24 and 31 weeks’ gestation were recruited from a rural prenatal clinic in the Mississippi Delta, USA	Retention rates were very high, and the study proved the feasibility of conducting preventative intervention on rural pregnant African American women. Mean number of intervention sessions attended was 4.58 (SD = 4.95, mode was 5.00) There was no significant difference across time 3 months after delivery between intervention and TAU for parent stress levels Depressive symptoms significantly dropped for the ROSE program group across time F(3, 39) = 4.44, *p* < 0.009, d = 0.26	Effectiveness of ROSE program in improving postpartum functioning in a group of African American pregnant women in a low-income rural area	The results were based on a small sample in rural South and may not be generalisable to other regions. Degree of depressive symptoms was assessed as opposed to the presence of postnatal depression, and therefore, the clinical status remains unknown
El-Mohandes et al. [34]	A randomized controlled trial to evaluate the efficacy of a cognitive behavioural intervention delivered during pregnancy in reducing behavioural risks in the postpartum period. The risks addressed included depression etc	2913 women were recruited prenatally and on average 10 weeks postpartum in six prenatal care sites in the District of Columbia	The intervention group was more successful in resolving all risks (47% compared with 35%, *p* = .007, number needed to treat = 9, 95% confidence interval [CI] 5–31) In resolving some risks (63% compared with 54%, *p* = .009, number needed to treat = 11, 95% CI 7–43) as compared with the usual care group Women in the intervention group were more likely to resolve all risks (odds ratio 1.86, 95% CI 1.25–2.75, number needed to treat = 7, 95% CI 4–19) and resolve at least one risk (odds ratio 1.60, 95% CI 1.15–2.22, number needed to treat = 9, 95% CI 6–29)	An integrated multiple risk factor intervention addressing psychosocial and behavioural risks delivered mainly during pregnancy can have beneficial effects in risk reduction at postpartum period	The delivery of the postpartum booster sessions was limited to one to two sessions only, which may not have been adequate, especially in the case of depression. The study’s decision to exclude anxiety as a targeted risk factor may also have affected the efficacy of the intervention. There was also a high rate of loss to follow-up (20% of participants)
Grote et al. [41]	Pre-/post-test to examine whether culturally relevant brief interpersonal psychotherapy (IPT-B) confers greater advantages to low-income pregnant women than those that accrue from enhanced usual care in treating depression for the sample population	53 mothers who were 10 to 32 weeks’ gestation recruited from a large public hospital in Pittsburgh, USA. 33 participants in the sample were African American	Participants in the IPT-B group over time were feeling significantly less depressed with large effect size (χ^2^ = 9.06, df = 1, *p* < .003; Cohen's h = .96), anxious and spending more enjoyable contact with friends and time in exciting activities compared to a usual care group Mothers in the IBT-B group more likely to report they were doing an excellent job in taking care of their babies needs and engaging in physical contact and play with their baby compared to control group (1.47 ± .18 vs 1.78 ± .26 respectively (t = 4.47, df = 42, *p* < .001, d = 1.35) In all areas, effect sizes became stronger as more time passed from baseline	Enhanced IPT-B compared with enhanced usual care showed a significant reduction in depression diagnoses and symptoms and social functioning up to 6 months postpartum in mothers	Small sample size, participants in the usual care group were more difficult to reach than participants in the intervention; these apparent differences made the study raters less likely to remain blind which poses a threat to internal validity
Holditch-Davis et al. [32]	Premuted Block Randomised Controlled trial to examine the effects of the auditory-tactile-visual-vestibular (ATVV) intervention and Kangaroo Care (KC) on maternal distress and the mother-infant relationship compared to an attention control group	240 preterm infants who weighed less than 1750 g and their mothers were recruited from 4 hospitals, USA. 64.1% of ATVV mothers, 64.2% of KC and 76.5% of control mothers were African American	KC mothers showed a rapid decline in worry than ATVV or control group Mothers who engaged in any intervention was associated with lower parenting stress compared to mothers who did not engage in any intervention (a form of massage only-t(195) = − 3.33, *p* < 0.001; KC only-t(195) = − 2.90, *p* < 0.01; both-t (195) = − 2.66, *p* < 0.01)	Findings suggest that short-term interventions have important effects on mothers and their preterm infants, particularly in the first half of the first year	Limited sample size limits examining other factors that could impact parenting and maternal distress. Positive short-term effects, long term effects not as significant
Jesse et al. [43]	A longitudinal study to explore the feasibility and efficacy of a 6-week culturally tailored cognitive-behavioural intervention offered to rural, low income women at risk of antepartum depression	146 between 6- and 30-weeks pregnant low-income women recruited, USA	The cognitive-behavioural intervention significantly reduced scores for depressive symptoms for African American women at high risk (n = 43 from baseline to posttreatment (5.59 vs 2.18, *p* = .02) and from baseline to one-month follow-up (6.32 vs 3.14, *p* = .04) For low-moderate risk African American women, the mean reduction in Beck's Depression Inventory-II scores significantly reduced from baseline and posttreatment compared to treatment as usual group (5.20 vs .70; *p* = .02)	The study proves the feasibility to screen, recruit and enrol rural low-income women who are at risk of postnatal depression. The study highlights the importance of integrating cognitive-behavioural intervention in the local health department to reach the at-risk, underserved group	The study only recruited low-income participants at risk for depression and therefore, cannot be generalised to other groups
Jesse et al. [42]	A pilot study aimed at reducing the risk for antepartum depression (APD) among African American and Caucasian women in a public health prenatal clinic	63 participants at risk of ADP. Of the 63 participants, 26 met all inclusion criteria. 17 women completed all six intervention sessions	94% of participants who completed their 1-month post-intervention interviews had an antepartum recovery rate of 81% (13/16, EPDS ≤ 10) Participants reported that various aspects of the program were helpful, and they continued to use the intervention exercises after the end intervention	The brief culturally tailored cognitive behavioural intervention for African American and Caucasian rural low-income women at risk of APD was feasible, effective, and helpfulness	Small sample size with only 17 women who completed all the six intervention sessions
Mendelson et al. [25]	A randomised controlled trial to examine the intervention impact on 3 proximal outcomes that are theoretically linked with the interventions model of change and have been empirically linked with risk for depression: mood regulation, expectancies, perceived social support and coping	78, low-income perinatal women recruited from one of four home visitation programs in Baltimore City, USA	The intervention group from baseline to 6-month follow-up in the intervention group experienced 16% greater gain in mood regulation compared to the usual care group (β = 0.16, SE = 0.03, *p* < 0.001) Growth in perceived social support from baseline to 6-month follow-up was 6.66 points greater in the intervention group compared to control group (β = 0.14, SE = 0.07, *p* < 0.05) Surprisingly, the intervention group had a 14% greater increase in avoidant coping strategies between baseline and 6-month post-intervention follow up compared to the control group (β = 0.14, SE = 0.07, *p* < 0.05)	The Mothers and Babies course enhances mood regulation in participants and may facilitate the prevention of depression over time. The study is one of the few trials that were of a randomised controlled design and longitudinal assessments with 3 month and 6-month follow-ups	The study was powered to detect group differences in depressive symptoms, not in the reported proximal outcomes. The small sample size may have limited the ability to detect small effects
Lenze and Potts [44]	A randomized controlled trial for brief interpersonal psychotherapy for depression during pregnancy in a low-income population. Aimed to replicate Grote et al. [41] brief-IPT model using similar modifications to engage low-income women into treatment	Pregnant women, aged ≥ 18, between 12- and 30-weeks’ gestation were recruited from an urban prenatal clinic. Women scoring ≥ 10 on the Edinburgh Depression Scale and meeting depressive disorder criteria were randomized to either brief-IPT (n = 21) or ETAU (n = 21)	After controlling for concurrent depressive symptoms, depression scores significantly decreased in both brief-IPT and ETAU Brief-IPT participants reported significant improvements in social support satisfaction as compared to ETAU participants Brief-IPT participants reported high satisfaction with the program	Brief-IPT for perinatal depression is acceptable to low-income women and is helpful for improving depressive symptoms and social support	The interpretation of study results is limited due to small sample size, use of self-report measures, and lack of an active psychotherapy control group. Many participants did not participate in the full 9-session course of treatment (average sessions attended = 6, range 0–17)
Logsdon et al. [31]	To test the effectiveness of an internet-based depression intervention on seeking depression treatment	151 adolescent African American mothers who had given birth in the last year were recruited, USA	Being depressed (OR = 2.15, 95% CI 1.15–4.04, *p* = 0.005) and being exposed to the intervention (OR = 1.65, 95% CI 1.06–2.32, *p* = 0.012) increased odds for seeking treatment Intervention independently improved attitudes (B = 2.92, *p*-0.018), beliefs of perceived control (B = 2.06, *p* = 0.012), intention to see treatment (B = 2.00, *p* = < 0.001), and actually receiving treatment (B = 5.02, *p* < 0.001)	Internet-based intervention is an inexpensive method of increasing rates of treatment for depression in adolescent mothers	Self-reporting measures were used. Medical records were not available to report the participants' use of mental health services
Toth et al. [33]	Randomised Controlled Trial to evaluate the efficacy of interpersonal psychotherapy (IPT) for ethnically and racially diverse, economically disadvantaged women with major depressive disorder	Out of a total of 128 women, 59.4% were Black and 21.1% Hispanic low-income urban women with a 12-month-old infant, USA	IPT participants showed significantly greater decrease in depression over time compared to the enhanced community care group (ECT) B = − 4.483 (1.602), t = − 2.799, *p* = .005, d = − 0.519 There were significant changes in perceived stress favouring IPT B = − 0.196 (0.094), t = 2.078, *p* = .038, d = 0.51 IPT predicted increases in family social support compared to ECT A = 0.169 (0.088), t = 1.925, *p* = .054, d = 0.45)	The study demonstrated the efficacy of IPT for decreasing depression in a group of a low-income ethnically diverse group of women. The findings contribute to the importance of addressing the reluctance of low income and minority populations to access mental health services	A significant number of participants randomised to IPT declined treatment which resulted in a smaller number of participants in the sample
Sampson et al. [35]	A pilot study to test the feasibility and pre-test to post-test outcome of a Problem-Solving Therapy (PST) Intervention for Low-Income, Pregnant Women at Risk for Postpartum Depression	All participants were unemployed African American women. 85% were unmarried 61% had other children besides this pregnancy	The intervention had a 93% retention rate There were statistically significant improvements on measures of depression symptoms after intervention Participants had 100% completion of homework A decrease in depression scores from pre-test to post-test indicates promise for effectiveness of the intervention	Findings demonstrates the feasibility of implementation of a home visiting intervention for Postpartum Depression (PPD) in a community-based agency and provides the grounds for optimism about the effectiveness of a PST intervention for low-income women at risk for postpartum depression	There were some chances of social desirability bias due to the self-report nature of questionnaire and familiarity of culture matched of participants and caseworkers. Inability of the study to measure treatment fidelity by caseworker with planned approach of listening to recorded sessions
Zhang and Emory [36]	Randomised controlled pilot study which involved a 2 × 3 mixed model design, comparing treatment as usual (TAU) with the Mindful Motherhood intervention on several outcomes at pre-intervention, post-intervention, and 1-month post-intervention	65 African American women (31 = TAU; 34 = Mindful Motherhood)	Findings support the efficacy of the Mindful Motherhood training in reducing depressive symptoms, reducing reactive cortisol response, reducing pregnancy related stress and improving levels of mindfulness at post-intervention and improving pregnancy related positive experience and at 1-month follow-up	The study supports the efficacy of mindfulness-based interventions with African American women and encourage efforts to optimise recruitment and retention of underprivileged population	None of the intervention effects appeared to have lasting impact on the participants, and treatment did not appear to impact perceived stress or baseline salivary cortisol levels

Examples of studies excluded which were either mostly Caucasians, interventions not focused on parental well-being/depression; without a mother and/or child outcomes includes (1) Morrell et al. [29] who examined the clinical effectiveness of health visitor training in psychologically informed approaches for depression in postnatal women, (2) Horowitz et al. [30] employed nurse home visits to improve maternal/infant interaction and decrease the severity of postpartum depression.

## Discussion

### Enhance parenting confidence and self-care

Findings from the reviewed studies showed that maternal depression and other mental health conditions could be best treated with interventions designed to enhance parenting confidence and self-care [25, 26, 31–36]. This notion of the intervention was found to be effective across eight studies. For example, Crockett et al. [26] examined the feasibility, effectiveness and acceptability of an intervention program called Reach Out, Stand Strong: Essentials for New Moms (ROSE) for rural, low-income African American pregnant women who were at risk of postpartum depression. The program entailed interpersonal therapy that enhanced familial communication, social support, managing the transition, and focusing on factors associated with major depressive symptoms and perinatal depression. Women in the ROSE condition showed significant improvement in their depressive symptoms and parenting adjustment at 3 months postpartum than women in the treatment as usual condition.

In a randomised controlled trial (RCT) of skill-based intervention that empowers women at risk of postpartum mental health distress, Mendelson et al. [25] measured how Mother-Baby (MB) course could be adopted as a preventive measure for depression and the impact of perinatal depression on coping, social support and mood regulation among African American women. An MB course is a manualised skill-development training material blended with core elements of cognitive behavioural therapy to prevent the risk of perinatal depression among ethnically diverse and low-income mothers [37]. This was delivered by trained facilitators who taught mothers how to harness and utilise enjoyable and supportive contacts, modulate their negative automatic thoughts and increase pleasant activities. Findings showed 16% greater prevention of depression over time and more effective mood regulation from baseline to 6 months follow up in the intervention group compared to the control group. These findings have been supported in previous research on the effectiveness of the MB course and its ability to prevent depressive symptoms and other risks of mental health problems in mothers [37, 38]. Crockett et al. [26] and Mendelson et al. [25] illustrated maternal programmes that encapsulate skills-oriented intervention, enhancing parenting confidence and self-care in postpartum periods and beyond.

### Effective mother–child interpersonal relationship

Mother–child relationship has significant implications for maternal mental health, and therefore, the current review showed recurrent emergence of interventions targeting effective mother–child relation [25, 31, 32]. For instance, Holditch-Davis et al. [32] examined the mother-infant relationship and its effects on maternal mental distress using a maternally administered intervention designed explicitly for preterm infants in the neonatal intensive care unit (NICU). Two interventions were compared; (1) Kangaroo Care and (2) Auditory, Tactile, Visual, and Vestibular (ATVV) interventions. The effectiveness of these two interventions on the mother-infant relationship and maternal mental distress were evaluated and compared with an attention control group. Findings showed a rapid decline in maternal depressive symptoms and lower parenting stress across all intervention groups with higher scores on the Home Observation for Measurement of the Environment (HOME) inventory. HOME is a scale that measures the quality and quantity of support available in the child's home environment [39]. ATVV is an intervention that involves a series of activities with an infant such as moderate stroking, talking, rocking and keeping eye-contact [40]. Whereas, Kangaroo intervention entailed a process of holding an infant to ensure skin-to-skin contact around the mother’s breasts [32].

Mendelson et al. [25] also focused on the mother–child interpersonal relationship in their study, in which the Mother and baby (MB) Course was adopted as the basis for preventing maternal depression. In this intervention, "the mother-baby relationship is emphasised throughout the course, including ways to enhance parenting practices using course skills" [25  p. 212]. The MB course involves core elements of cognitive behavioural intervention that translates into quality mother–child relationship and has consistently shown to reduce maternal depression, especially among low-income women [37, 38].

### Culturally appropriate maternal care

Previous studies have shown low engagement and high dropout rates of psychological treatment among ethnic minority populations in HICs compared to the native population which suggests that current treatment options lack cultural relevance or appropriateness [14, 16]. Therefore, cultural beliefs, values and traditions are essential in intervention planning and execution for addressing maternal depression [25, 26, 33, 35, 41–44]. In the Grote et al.’s [41] study using an RCT design, culturally relevant and enhanced brief Interpersonal Psychotherapy (IPT-B) had a greater effect on treating depression in low-income pregnant women compared to those receiving enhanced usual care. Enhanced IPT-B consists of engagement sessions and later followed by eight IPT-B sessions covering up to 6 months postpartum period.

Most importantly, the culturally relevant materials were embedded to augment the intervention. For example, the engagement session was based on ethnographic and motivational interviewing designed to build rapport and trust to address the practical aspects of lived experiences of cultural and psychological barriers to mother and childcare among low-income depressed women. Therefore, the practitioners approached the mothers with the core elements of culturally sensitive principles of ethnographic interviewing in which the interviewer employs the role of a learner to understand the women from their cultural perspectives without any bias, focusing on; unconditional appreciation of participants’ cultural values, beliefs and traditions in terms of their views about depression, health beliefs, coping practices or health-seeking behaviours. Women were asked what they would like to achieve during the intervention phase. Issues such as the importance of race, ethnicity and spirituality were put forward in the engagement sessions [41].

Considering the low-income and economically disadvantaged predicaments of the depressed women in this minority group, a pragmatic approach was integrated with the Enhanced IPT-B intervention to cater for basic needs (e.g. access to food and free bus passes and bay supplies) and to facilitate access to sustainable social services (e.g. effective childcare, job training and access to housing) which were included as core aspects of the intervention delivery. Findings from the Enhanced IPT-B intervention highlights its effectiveness in reducing antenatal depression, and further improving social functioning and preventing relapse in the postpartum period of up to 6 months.

Furthermore, two studies implemented reinforcement strategies, weekly from their home visitor [25], and the other weekly calls and/or texts about the upcoming session [27]. A combination approach is essential to be considered given the numerous obstacles that may hinder African and Caribbean women from engaging in treatment, especially when they are from low-income backgrounds. Translated materials and topics such as spiritual-related resources were added to the culturally adapted intervention [43].

### Internet-mediated care for maternal distress

Modern technological innovation now plays a significant role in the mental health healthcare provision. One of the overarching themes in the present review is internet-mediated care for maternal depression [27, 31]. For example, Logsdon et al. [31] examined the effectiveness of the internet-based approach to treating depression among African American adolescent mothers. The intervention included vignettes, resources, questions and answers developed based on the theory of planned behaviours (TPB). Immediately after the intervention and 2 weeks post-intervention, participants (n = 151) responded to questions on depressive symptoms based on TPB variables. They completed the intervention from any computer device of their choice. This was compared with the control group (n = 138) which entailed visits to homes and community organisations.

The intervention led to significant improvements in perceived control on maternally related self-care, positive attitude change, increased intention to seek help, and active behaviours for seeking actual treatments. Other advantages accrued to the internet-based intervention were about the cost of delivering it, which was least expensive with reduced costs of logistics and time, which may be especially beneficial for mothers with limited resources. A similar study, Boyd et al. [27] conducted a pilot RCT using social media for postpartum intervention for depressed low-income mothers. Groups were compared using social media (Facebook) and in-person interventions for African American mothers who screened positive with postpartum depression from paediatric clinics. Both interventions employed the parents interacting with infants (PIWI) which involved eight weekly sessions including behaviour activation and depression psychoeducation, parent–child interaction such as play, laughter and safety. Findings showed that women in the social media intervention group had lower depressive symptoms than women in the in-person.

Both Logsdon et al. [31] and Boyd et al. [27] studies showed the potential effectiveness of using online facilities for reducing maternal depression. Their findings have been supported in the previous literature. For example, online intervention to improve positive parenting behaviours and ameliorate child-related behavioural problems [45], treatment of depression, improve attitudes about treatment, relapse prevention among young mothers [46, 47], and improved parenting skills and infant feeding [48].

This study was conducted shortly before the Covid-19 pandemic era; however, the unequal distribution of infection and death rates of the Covid-19 pandemic among African and Caribbean populations in HICs has exposed longstanding systemic issues of racial inequalities [19]. For example, in the UK, those who experienced higher societal disadvantages faced more significant health risks [49]. Reports have shown that there have been between a 10% and 50% higher risk of death among African and Caribbean populations compared to the White British population [49]. Considering the current pandemic, which may further alienate already marginalised people, there is an urgency to understand the effectiveness and engagement of culturally adapted online help or ‘tele-intervention’ delivered remotely for these ethnic groups.

## Limitations and suggestions for future research

The review explored psychological intervention for depressed African and Caribbean mothers in HICs. However, the review outputs were limited to a few studies (N = 13) that met the inclusion criteria and captured various North America interventions only. This implies that women of African and Caribbean origins are disproportionately affected by maternal depression and distress [1, 3–5]. Yet, there are limited evidence-based interventions for maternal distress that are specifically designed and made culturally appropriate for African and Caribbean mothers. Culturally relevant psychological interventions are essential for maternal depression [8, 41]. Therefore, the present review recommends culturally adapted interventions for mothers of African and Caribbean origin living in HICs. In-person contact and online intervention studies are required to establish a nexus of evidence-based resources for usability in maternal health-related policies and clinical practice for these mothers.

Following on from the review of psychological interventions for maternal depression, the direction of the future psychological intervention—a Learning Through Play plus Culturally adapted Cognitive Behaviour Therapy (LTP + CaCBT) [50] may be useful for African and Caribbean depressed mothers living in HICs. LTP + CaCBT intervention package is manualised and piloted in Kilifi, Kenya and can be delivered by community health trained workers not requiring mental health experts [51]. The programme enables mothers to improve their children psychosocial development by engaging in mother–child play. The theoretical underpinnings of LTP are found in Piaget's theory of cognitive development [52] and Bowlby’s attachment theory [53, 54]. The SickKids Centre for community mental health (CCMH) Learning Institute illustrated LTP as a low-cost intervention with culturally appropriate homemade toys, books, and materials commonly found in the homes and used to promote cultural relevance and keep costs to the minimum [55]. The CaCBT instructions for each session are organised into modules that focus on feelings, thoughts, and behaviours with specific interventions focusing on psychoeducation, problem-solving, managing negative thoughts, behaviour activation, and relaxation [34, 56].

The essential aspects of LTP + CaCBT were identified in the reviewed interventions. For example, Mendelson et al. [25] MB course focused on mood regulation, perceived social support, and coping. Similarly, the Auditory, Tactile, Visual, and Vestibular (ATVV) interventions which involve series of activities with an infant such as moderate stroking, talking, rocking and keeping eye-contact [40], appeared to capture some elements of LTP. Both LTP and ATVV seem to stimulate mother–child attachment and positive outcomes.

Our recommendation for culturally adapted psychological interventions might pave the way for preliminary efforts towards decolonising mental health provision in HICs. Other essential aspects of psychological intervention that could be integrated for these mothers may include their religion, philosophy, spiritualism, ritualism that are more ‘organic’ to the African and Caribbean people and culture.

The study’s originality and significance are delineated in the paucity of research concerning depressed African and Caribbean mothers in HICs who are often neglected. Thus, the proposed LTP+CaCBT can offer a paradigm shift in culturally appropriate mental healthcare for African and Caribbean mothers. Finally, the researchers explored the possibilities of conducting a meta-analysis on the trials included in the present review; however, the trials included were mostly (a) pilot studies that tested treatment feasibility (b) various outcome measures with no fixed-effect, and therefore, appeared incompatible for a meta-analysis. Future reviews could be designed to perform an in-depth meta-analysis that could yield a more precise estimate of true effect outcomes [57].

## Conclusion

The legacies of slavery, colonialism, and racism still impact African and Caribbean people’s mental health experiences in unique ways, unlike any other ethnic people in HICs. Maternal experiences are emotionally demanding considering the delicate nature of care required during the prenatal, perinatal or postnatal periods. Due to insufficient culturally sensitive care, maternal depression is more challenging among African and Caribbean mothers living in HICs. The present review has synthesised intervention studies for maternal depression among women of African and Caribbean origin. The review also highlights that there is limited literature on maternal depression and culturally appropriate interventions. However, our proposal for culturally adapted psychological interventions (e.g. LTP + CaCBT) could serve as part of the preliminary efforts to decolonise mental health provision in HICs. Both in-person contact and online culturally relevant studies are recommended for African and Caribbean mothers as the basis for building culturally relevant evidence-based resources for use in health policy initiatives and clinical practice.

## Supplementary Information

**Additional file 1.** Showing the risk assessment of each study.

## Data Availability

Not applicable.

## Abbreviations

ASSIA: Applied social science index and abstracts
ATVV: Auditory, tactile, visual, and vestibular
CaCBT: Culturally adapted cognitive behaviour therapy
CCMH: Centre for Community Mental Health
HIC: High-income countries
HOME: Home observation for measurement of the environment
IPT-B: Interpersonal psychotherapy
LTP: Learning through play
MB: Mother baby
NICE: National Institute for Health and Care Excellence
NICU: Neonatal intensive care Unit
PICo: Population, phenomenon of interest, and context
PIWA: Parents interacting with infants
PRISMA: Systematic reviews and meta-analyses
PROSPERO: Prospective register of systematic reviews
RCT: Randomised controlled trial
ROSE: Reach out, stand strong: essentials for new moms
SPUR: Scholarship projects for undergraduate researchers
TPB: Theory of planned behaviours
