# Supplementary material for: Psychological interventions for maternal depression among women of African and Caribbean origin: a systematic review
Source: BMC Womens Health. 2021 Feb 26;21:83. doi: 10.1186/s12905-021-01202-x (PMC7907308; doi:10.1186/s12905-021-01202-x)
Supplement: Supplementary file 1 — Additional file 1. Showing the risk assessment of each study. [file 12905_2021_1202_MOESM1_ESM.docx]

**Conflict of bias within studies:** *The* QualSyst assessments criteria which consisted of 14 questions concerning bias for each article was adopted. The appraisal of each study was done separately by creating a table which adopted a point scoring system (See Appendix).

|  |
| --- |
|  |

**Quality assessment**: The review assessed each article for bias using a table which contained a score for the 14 questions contained in the QualSyst assessment. Yes was scored 2 points, no was accorded 0 and partial was scored at 1. The points were cumulatively added up to 28, and where possible, in some studies where points were not applicable this was deducted. The adoption of this process ensured against risk of bias that may have limited the inclusion of relevant studies in informing the review’s conclusion.

The total sum based on the scoring were calculated using the following format (number of “yes” * 2) + (number of “partials”*1) + (number of “no” 0) against a total possible sum = 28

# The QualSyst Quality Assessment

| **1** | Question / objective sufficiently described? |
| --- | --- |
| **2** | Study design evident and appropriate? |
| **3** | Method of subject/comparison group selection *or* source of information/input variables described and appropriate? |
| **4** | Subject (and comparison group, if applicable) characteristics sufficiently described? |
| **5** | If interventional and random allocation was possible, was it described? |
| **6** | If interventional and blinding of investigators was possible, was it reported? |
| **7** | If interventional and blinding of subjects was possible, was it reported? |
| **8** | Outcome and (if applicable) exposure measure(s) well defined and robust to measurement / misclassification bias? Means of assessment reported? |
| **9** | Sample size appropriate? |
| **10** | Analytic methods described/justified and appropriate? |
| **11** | Some estimate of variance is reported for the main results. |
| **12** | Controlled for confounding? Variables – gender take out diagnose about gender age culture |
| **13** | Results reported in sufficient detail. |
| **14** | Conclusions supported by the results. Yes |

# Appendix: The QualSyst Quality Assessment:

| **Author** | **Scores** | **Scores** | **Scores** | **Scores** | **Scores** | **Scores** | **Scores** | **Scores** | **Scores** | **Scores** | **Scores** | **Scores** | **Scores** | **Scores** | **Total score** |  |
| --- | --- | --- | --- | --- | --- | --- | --- | --- | --- | --- | --- | --- | --- | --- | --- | --- |
|  | **1** | **2** | **3** | **4** | **5** | **6** | **7** | **8** | **9** | **10** | **11** | **12** | **13** | **14** |  | |
| Boyd et al (2019) | 2 | **2** | **2** | **2** | **2** | **2** | **1** | **2** | **2** | **2** | **1** | **2** | **2** | **2** | **26** | |
| Crockett et al (2008) | 2 | 2 | 2 | 2 | 2 | 1 | 2 | 2 | 1 | 2 | 2 | 2 | 2 | 2 | 26 | |
| El-Mohandes et al. (2008) | 2 | 2 | 2 | 2 | 2 | 1 | 2 | 2 | 2 | 2 | 2 | 2 | 2 | 1 | 26 | |
| Grote et al., 2009 | 2 | 2 | 2 | 2 | 2 | 1 | 2 | 2 | 1 | 2 | 2 | 2 | 2 | 2 |  | |
| Holditch-Davis et al. (2014) | 2 | 2 | 2 | 2 | 2 | 2 | 2 | 2 | 1 | 2 | 2 | 2 | 2 | 2 | 27 | |
| Jesse et al., 2015 | 2 | 2 | 2 | 1 | 1 | 2 | 2 | 1 | 2 | 2 | 2 | 2 | 2 | 2 | 25 | |
| Jesse et al., 2010 | 2 | 2 | 2 | 2 | 2 | 2 | 2 | 2 | 1 | 2 | 2 | 2 | 2 | 2 | 27 | |
| Mendelson et al., 2013 | 2 | 2 | 2 | 2 | 2 | 2 | 2 | 2 | 1 | 2 | 2 | 2 | 2 | 2 | 27 | |
| Lenze & Potts (2017) | 2 | 2 | 1 | n/a | n/a | n/a | n/a | n/a | 1 | 2 | 2 | 2 | 2 | 2 | 16 | |
| Logsdon et al., 2018 | 2 | 2 | 2 | 2 | 2 | 2 | 2 | 2 | 2 | 2 | 2 | 2 | 2 | 2 | 28 | |
| Toth et al., 2013 | 2 | 2 | 2 | 2 | 2 | 2 | 2 | 2 | 2 | 1 | 2 | 2 | 2 | 2 | 27 | |
| Sampson et al., 2016 | 2 | 2 | 2 | 2 | 1 | 2 | 2 | 2 | 2 | 2 | 2 | 2 | 2 | 2 | 27 | |
| Zhang & Emory, 2015 | 2 | 2 | 2 | 2 | 1 | 1 | 2 | 2 | 2 | 2 | 2 | 2 | 2 | 2 | 26 | |
| **Summary of scores** |  |  |  |  |  |  |  |  |  |  |  |  |  |  |  | |
